# Weber’s Law, the Magnitude Effect and Discrimination of Sugar Concentrations in Nectar-Feeding Animals

**DOI:** 10.1371/journal.pone.0074144

**Published:** 2013-09-10

**Authors:** Vladislav Nachev, Kai Petra Stich, York Winter

**Affiliations:** 1 Humboldt University, Berlin, Germany; 2 Bielefeld University, Bielefeld, Germany; Universität Bielefeld, Germany

## Abstract

Weber’s law quantifies the perception of difference between stimuli. For instance, it can explain why we are less likely to detect the removal of three nuts from a bowl if the bowl is full than if it is nearly empty. This is an example of the magnitude effect – the phenomenon that the subjective perception of a linear difference between a pair of stimuli progressively diminishes when the average magnitude of the stimuli increases. Although discrimination performances of both human and animal subjects in various sensory modalities exhibit the magnitude effect, results sometimes systematically deviate from the quantitative predictions based on Weber’s law. An attempt to reformulate the law to better fit data from acoustic discrimination tasks has been dubbed the “near-miss to Weber’s law”. Here, we tested the gustatory discrimination performance of nectar-feeding bats (*Glossophaga soricina*), in order to investigate whether the original version of Weber’s law accurately predicts choice behavior in a two-alternative forced choice task. As expected, bats either preferred the sweeter of the two options or showed no preference. In 4 out of 6 bats the near-miss to Weber’s law provided a better fit and Weber’s law underestimated the magnitude effect. In order to test the generality of this observation in nectar-feeders, we reviewed previously published data on bats, hummingbirds, honeybees, and bumblebees. In all groups of animals the near-miss to Weber’s law provided better fits than Weber’s law. Furthermore, whereas the magnitude effect was stronger than predicted by Weber’s law in vertebrates, it was weaker than predicted in insects. Thus nectar-feeding vertebrates and insects seem to differ in how their choice behavior changes as sugar concentration is increased. We discuss the ecological and evolutionary implications of the observed patterns of sugar concentration discrimination.

## Introduction

The capacity of decision-makers to make choices that maximize profitability crucially depends on their ability to sense and evaluate differences among alternative choice options [1]–[3]. When sequential sampling of multiple options takes place, the comparison of stimuli is assumed to occur on cognitive representations of physical scales [4]–[6]. As direct observations and measurements of subjective sensations are not possible, the relationships between the physical and the psychological scales are studied by measuring behavioral output or neuronal activity. A well-known and highly discussed psychophysical invariant is Weber’s law [1], [4], [6], [7], which states that judgments of stimulus change are at a constant proportion of stimulus magnitude (e.g. the length of a line measured in mm, a time interval measured in seconds, or the concentration of a sugar solution measured in % weight/weight, etc.). The differential threshold has been defined as the smallest difference between two stimuli that an observer is able to detect. More recently, this definition has been extended to refer to the difference between two stimuli that an observer can detect with a certain probability [8]–[12]. In experiments, the magnitude of one of the stimuli is usually kept fixed throughout a block of trials, and this stimulus is called the standard stimulus, whereas the second stimulus is called the referent stimulus. Weber’s law can thus be expressed using the Weber fraction:
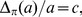
(1)where *a* is the magnitude of the standard stimulus, Δ_π_ (*a*) is the differential threshold between the standard and a referent option at probability *π*, and *c* is a constant that depends on the observer and the sensory modality. In experimental settings the probabilities *p*(*x*,*a*) (*discrimination performances*) that a stimulus with a magnitude *x* is judged greater than a stimulus with magnitude *a* are measured usually in two-alternative forced choice (2AFC) or similar tasks and form the so-called psychometric function *P_a_*(*x*): = *p*(*x*,*a*). We now define the *relative intensity* of two stimuli *x* and *a* (*x*>*a*) as



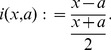
(2)If Weber’s law holds then an observer encountering two sugar concentration pairs with the same relative intensity should select the higher concentration stimulus with the same probability in each pair of concentrations.

In a choice between two magnitudes discrimination performance usually improves as the difference between the options increases (distance effect) and it usually declines as distance (the absolute difference between the two options) is kept constant but the average magnitude of the two options increases (magnitude effect). Relative intensity and the Weber fraction are both ratios that combine these two effects. Mathematicians have demonstrated that as long as discrimination probabilities are determined by differences in psychophysical scale values, the choice of measurement of the physical scale is immaterial [8]. The advantage of relative intensity over the Weber fraction is that it can also be used in situations where the dichotomy between standard and referent option does not apply. For example, when the options are presented simultaneously without cues that can be used to identify a standard option across test conditions, both of the stimuli can be seen as a standard.

Psychometric functions are usually assumed to be sigmoid functions such as the distribution functions of the normal, logistic, Weibull, and Gumbel distributions [13]–[16]. The Weibull function has the advantage that it can be parameterized in terms of biologically meaningful parameters, the threshold, slope, and lapse rate [13]–[17]. The point on the curve halfway between the lower and upper asymptote (corresponding to a discrimination performance of ca. 75%) is referred to as the threshold. The slope of the function at the threshold can be interpreted as a reliability measure of sensory performance [16]. An important distinction needs to be made at this point between discrimination performance and the capacity to discriminate. The actual measured discrimination performance is usually lower than expected, because of lapsing, i.e. making a decision that is not based on relative intensity but may constitute an error due to noise, motivational problems or other factors of non-perceptual nature, such as exploratory behavior. Foraging animals face the exploration-exploitation dilemma and need not always make choices based on expected values. In psychometric analyses it is assumed that an observer has a constant lapse rate, i.e. a constant probability to select an option not based on relative intensity but using an alternative rule. The lapse rate is calculated as one minus the upper asymptote of the psychometric curve multiplied by two.

### Near-Miss to Weber’s Law

Empirical tests of Weber’s law in the fields of acoustic [9], [18] and visual [11] perception have revealed that for very high stimulus magnitudes observers perform better than predicted. (Fechner [4] pointed out that the Weber fraction remains constant only for a limited range of stimulus magnitudes.) Discrimination performances in these studies are better fitted by an expression that allows sensitivity to grow as a power function of stimulus magnitude [9]–[12]:
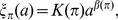
(3)where *K*(*π*) and *β*(*π*) are real valued parameters that may depend on the value of *π*, and *ξ_π_*(*a*) gives the magnitude of a stimulus that is judged greater than *a* with probability *π*. If the value of *β*(*π*) is one, then Weber’s law is satisfied. Equation 3 has been demonstrated to hold over a wide range of magnitudes and because the exponent *β* is typically estimated around 0.9, equation 3 is referred to as the near-miss to Weber’s law [9]–[12], [18].

Here we consider a slightly different formulation of the near-miss to Weber’s Law and define the *near-miss relative intensity* of two stimuli with magnitudes *x* and *a* with *x*>*a* as
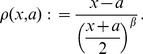
(4)


The parameter *β* determines how strong the magnitude effect is with respect to the distance effect and if it equals 1, then near-miss relative intensity reduces to relative intensity. Thus, we consider Weber’s law to be satisfied when the parameter *β* is estimated to be one and invoke the near-miss to Weber’s law when *β* significantly differs from one.

Knowledge of gustatory information processing [7], [19], [20] is important for understanding the formation of economical food preferences [21]–[24] and may have implications for our understanding of the co-evolution of nectar rewards and animals’ discrimination abilities. Diverse groups of nectar-feeding animals such as bees [24]–[26], birds [27]–[29] and bats [23], [30], [31] show a general pattern of preference for sweeter sugar solutions and more precise discrimination at low concentrations. (Discrimination of nectar volume rewards also follows this pattern, so that choice proportions in 2AFC tests can be fitted against the relative intensity of the stimuli [22], [32], [33].) Although the results were consistent with Weber’s law, the law was not rigorously tested in these studies. Here, we present a series of 2AFC tests with nectar-feeding bats (*Glossophaga soricina*) designed to test directly whether concentration pairs with the same relative intensity result in equal discrimination probabilities (as predicted by Weber’s law). We also tested whether near-miss relative intensity is a better predictor of discrimination performance than relative intensity. We used the method of constant stimuli with a standard feeder giving rewards with 20% weight/weight sugar concentration and a test (referent) feeder, whose concentration was systematically varied. This allowed us to construct individual psychometric functions for the discrimination of sugar concentration. Finally, we reviewed previously published data on bats, hummingbirds, honeybees, and bumblebees in order to test whether Weber’s law or the near-miss to Weber’s law provide a better fit to sugar concentration discrimination performance.

## Animals, Materials and Methods

### Ethics Statement

Treatment of the experimental animals complied with the national laws on animal care and experimentation, under license of Veterinäramt Bielefeld. A specific ethical approval was not required due to the observational nature of the study that caused no suffering, damage, or pain to the animals.

### Subjects

Experiments were carried out with five female and one male Pallas’s long-tongued bats (*Glossophaga soricina*) from the same colony at Bielefeld University. The climatic conditions in the housing room (ca. 2.2×3.4×3.7 m) were ca. 25°C and ca. 60% humidity. The colony received ad libitum 20% honey water, honey water mixed with supplementary nutrients: Nektar Plus (Nekton®, Günter Enderle, Pforzheim, Germany) and Alete2Folgemilch (Nestle), Multi-Mulgat® (BioWeyxin, Veyx-Pharma GmbH, Schwarzenborn, Germany) and bee-collected flower pollen. Once a month they were also provided with live flies (*Musca domestica*). The six experimental individuals were adult, older than one year of age. Bats were marked with unique Radio Frequency Identification tags (RFID: 12×2.1 mm, 124 kHz, Sokymat) using self-made silicon collars (total collar and RFID weight = 0.20 g, less than 2.5% of the body mass of the smallest bat). After the experiment, the animals were returned to the colony. Light conditions during the experiments were 12∶12 LD and all experiments were conducted during the dark phase.

### Cage and Feeder System

**Figure 1:**
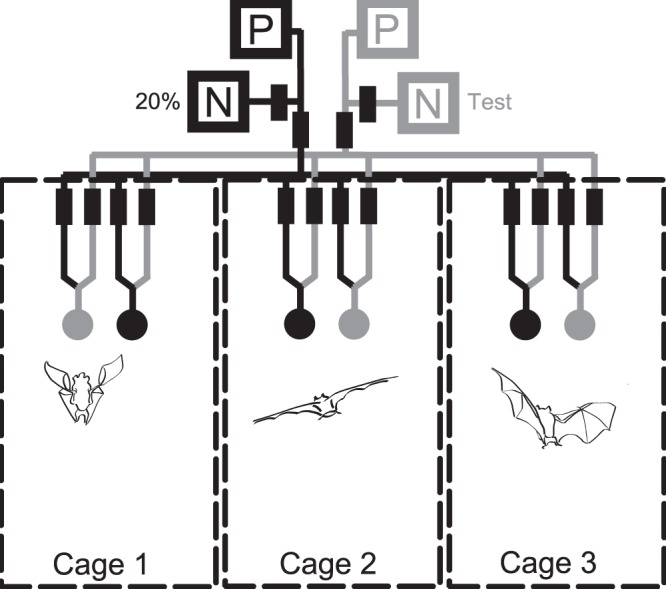
Schematic overview of the cage and feeder system. Two feeders (circles) were placed inside each cage (boxes with dashed outlines). Every feeder was connected via tubes (continuous lines) to two nectar pumping systems. One pump (black P) was connected to a 20% sugar solution reservoir (black N) and the other pump (gray P) was connected to the reservoir with the test concentration (gray N), which differed with test condition (Table 1). The flow of nectar was controlled with the pumps and pinch valves (black rectangles).

During the experiments each bat was placed individually inside one of three adjacent cages (0.7×1.5×2.2 m) [34], inside a 3.4×3.7×3.8 m room. Each cage contained two computer-controlled feeders on the back wall and a hanging roost. Visits to the feeders were automatically detected by infrared sensors. Upon detecting a visitor, feeders delivered a fixed amount of 20 µL sugar water (hereafter ‘nectar’) as a reward that the bats removed by licking while briefly hovering in front of a feeder. Nectar reward delivery was controlled by two syringe pumps using two gas-tight Hamilton glass syringes (Series 1025). Feeders were connected to the pumps via two identical systems of pinch valves and tubes (Figure 1). Access to each feeder could be blocked automatically by moving a swivel arm with a plastic guard in front of the feeder opening. Details of the experimental apparatus are given in [34].

Nectar consisted of equal parts of fructose, glucose, and sucrose dissolved in water, with a hexose to sucrose ratio similar to that in natural nectars of glossophagine-pollinated plants [35]. During a particular night one feeder in each cage received nectar from one pumping system, and the other feeder from the other system (Figure 1). System 1 was always filled with 20% w/w concentration and the concentration in System 2 varied throughout the experiment. Thus, during a single night the concentration offered at each feeder was fixed and did not change. In order to prevent bacterial and fungal growth inside the tubing systems, they were rinsed regularly with 70% ethanol and, or in addition to, water.

### Experimental Schedule

Experiments were conducted consecutively with two groups of three animals each and each group was subjected to a series of 2AFC tests. The first group of three bats participated in calibration tests of the cage system for six nights before actual testing began (Table S1). The three bats of the second group started with the experiment on the day of the transfer to the cages.

The two feeders in every cage gave different sugar concentration rewards during each experimental session. Every session lasted 12 hours and consisted of two phases: a forced alternation phase, and a choice phase. The alternation phase lasted for 100 visits (50 samplings per feeder) and ensured that the bats experienced both nectar concentrations equally. Strict alternation was achieved by blocking a feeder opening with the plastic guard after every visit. During the choice phase the plastic guards were automatically removed from both feeders until the end of the session so that bats could access both feeders freely. During the inter-session interval (ISI = 12 h) the lights were on and all feeders were inaccessible.

Of the two feeders in each cage one (standard feeder) always gave rewards with 20% w/w sugar concentration (632 mmol L^−1^ sucrose equivalents, [36]). The nectar concentration of the other feeder (test feeder) was systematically changed (Table 1) and ranged from 8 to 50% weight/weight (226 to 1796 mmol L^−1^ sucrose equivalents). We avoided concentrations higher than 50%, because for sugar concentrations above ∼52% the increase in viscosity is expected to cause a reduction in net energy gain [37], [38].The test concentrations were chosen to be symmetrical around the standard concentration of 20% with respect to their relative intensity value. The sequence of test concentrations within both series was random. However, every condition was presented twice on consecutive nights on which the feeder positions for the test and standard concentration were exchanged (Figure 1, black and gray feeders), as a control for positional biases. Since the cages were supplied with nectar from the same two pumping systems, the sequence of test conditions was equal for bats within the same group. In each cage, the choice of position for the test feeder on the first presentation of a particular condition was pseudorandom, with an equal number of left and right starting positions for the test concentration.

**Table 1:** Sequence of experimental conditions in the two subject groups.

	Group 1		Group 2	
Sequence^a^	Test concentration^b^	Relative intensity^c^	Test concentration^b^	Relative intensity^c^
1	12.5	0.46	20	**0.00** d
2	18.6	0.07	30	**0.40**
3	25	**0.22**	8	0.86
4	29	**0.37**	18.6	0.07
5	8	0.86	13.3	0.40
6	21.5	**0.07**	50	**0.86**
7	32	**0.46**	16	0.22
8	30	**0.40**	13.8	0.37
9	13.3	0.40	32	**0.46**
10	20	**0.00** d	29	**0.37**
11	16	0.22	17	0.16
12	50	**0.86**	25	**0.22**
13	23.5	**0.16**	23.5	**0.16**
14	13.8	0.37	21.5	**0.07**
15	17	0.16	12.5	0.46

a Each condition in the sequence was tested twice on two consecutive nights, with the position of the test and standard feeder exchanged. In Group 1, the experiment was interrupted for 4 days between sequence 14 and 15 and in Group 2 for 8 days between sequence 7 and 8.

b Sugar solution concentrations are given in % weight/weight. The concentration of the standard was always 20% w/w.

c Relative intensity is calculated as the absolute difference between the test and standard concentrations divided by the average of the concentrations.

d Numbers given in bold correspond to the HIGH data set (test concentrations equal to or larger than 20%). The rest of the numbers correspond to the LOW data set. The comparison with 20% was included in both data sets. As the R script for psychometric analysis did not accept the 0 intensity value, it was entered as 1.0×10^−6^ instead.

### Data Analysis

Analysis was limited to the first one hundred visits of the choice phase, in order to analyze choice after an equal number of samplings at both feeders. For each bat and each condition we calculated the *relative intensity* and *discrimination performance*. The relative intensity was calculated as the absolute difference between the two sugar concentrations, divided by the mean concentration (see Equation 2). As explained in the introduction, this measure of intensity is analogous to the Weber ratio of Δ_π_ (a)/a and captures the expectations that discrimination performance should increase with the difference (distance effect) and decrease with the mean magnitude of the two options (magnitude effect). Discrimination performance was calculated over the two presentations of the same condition as the number of visits to the higher sugar concentration feeder divided by the total number of visits (*N = *200). Data deposited in the Dryad Digital Repository: http://dx.doi.org/10.5061/dryad.0838c [39].

### Psychometric Analysis

The data sets of each animal were separated into two subsets: the HIGH set contained the comparisons with concentrations larger than or equal to the 20% standard (Table 1) and the LOW set contained the comparisons with concentrations smaller than or equal to the 20% standard (Table 1). Psychometric analyses were performed on the two data sets from each individual and Weibull psychometric functions were fitted using the Bayesian algorithm proposed by Kuss et al. [15] using R 2.10.1 [40]. As prior function for the lapse rate we chose a beta distribution (2;10). For the threshold we chose a normally distributed prior with a mean of 1 and a standard deviation of 0.5, and for the slope a log-normal prior with a mean of 2 and a standard deviation of 1. We performed 5000 Markov Chain Monte Carlo (MCMC) sampling runs with a leapfrog step size of 100. From the individual psychometric functions obtained using this method, we calculated the mean threshold, slope, and lapse rate. We then used paired t tests to compare the three parameters of the psychometric functions obtained for the HIGH and LOW data sets. The prediction based on Weber’s law was that there would be no differences between the parameters from the two sets.

### Weber’s Law vs. Near-Miss to Weber’s Law

In order to test whether the near-miss to Weber’s law provides a better fit to observed data than Weber’s law we individually fitted psychometric functions as Weibull sigmoid curves using the following equation [15], [23], [24]:
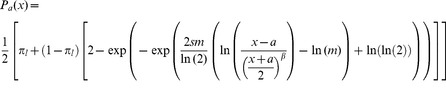
(5)where *x* is the larger and *a* is the smaller of the sugar concentrations of the test and standard feeders, *m* is the threshold, *s* is the slope at the threshold, *π_l_* is the lapse rate, and *β* is the exponent from Equation 4. In all models *x* and *a* were the independent variables, discrimination performance was the dependent variable, and *m*, *s* and *π_l_* were estimated parameters. Using Akaike Information Criterion (AIC) scores we compared non-linear models in which the parameter *β* was either fixed at one (reducing near-miss relative intensity to relative intensity) or estimated within the model. We used the non-linear least-squares function nls in R 2.15.0 [41].

### Reanalysis of Previously Published Data Sets

We reanalyzed data from sugar discrimination experiments with nectar-feeding bats, hummingbirds, bumblebees, and honeybees. When analyzing previously published work by other authors, we extracted numerical values from the published scatterplot figures using EasyNData [42] and converted the sugar concentration in percentage weight/weight units. In order to test whether the near-miss to Weber’s law provides a better fit to observed data than Weber’s law we analyzed the transformed data using the procedure described in the previous section. If the 95% confidence intervals for *β* in two different groups both spanned a convenient round number, this number was taken to be the *β* value of both groups. This was done because it is not otherwise possible to compare threshold and slope parameters for psychometric functions based on near-miss relative intensities with different *β* values. [Comparison of lapse rates can be done regardless of differences in *β* values.] Psychometric analyses on the different data sets were performed with Kuss’ algorithm [15] using as independent variables the near-miss relative intensity values calculated with the rounded *β* values.

### Nectar-feeding Bats

We used data from two-alternative free-choice experiments on a population of wild electronically tagged *G. commissarisi* bats foraging at a 6×4 array of computer-automated artificial flowers that recorded individual choices [23]. Two reward types were presented simultaneously, with half of the feeders delivering one reward type and the other half the second type. The sugar concentrations were in the range of 5 to 50% w/w. Asymptotic relative visitation rates to the higher concentration feeders were measured in 23 individuals. Here we analyzed the pooled data from all 23 bats. It has been demonstrated that this type of data pooling only causes an underestimation of the slope parameter, but does not affect the threshold and lapse rate [24]. The same pooling was done in the remaining analyzed studies as well, even if individual data were available, for better comparison between data sets.

### Hummingbird Data

Reanalyzed data were from a study on the concentration preferences in different hummingbird species [43]. The food intake from two adjacent feeders was monitored at half-hour intervals in nine individuals from five different species. The positions of the low and high concentration feeders were exchanged every half hour, for a total of 6 to 12 half-hour intervals. The sucrose concentrations were in the range of 0.15 to 1.10 M (5 to 33% w/w), with fixed differences of either 0.05 M (1.7% w/w), 0.10 M (3.4% w/w), or 0.20 M (6.7% w/w). Discrimination performance data were extracted from Figure 1 in [43].

### Bumblebee Data

We used data from two-alternative free choice experiments on two *B. impatiens* colonies containing some electronically tagged bumblebees foraging at an array of computer-automated artificial flowers [24]. In these experiments 10 blue and 10 yellow feeders were used, in a staggered checker-board formation, on a 5×2 array. Rewards were delivered with a probability of about 50% and the sucrose concentrations were in the range of 15 to 50% w/w. Discrimination performance was measured as the asymptotic relative visitation rates to the higher concentration feeders. Data were pooled over three marked individuals and an unknown number of unmarked individuals and analyzed together.

### Honeybee Data

Reanalyzed data were from a study on the concentration preferences in the Italian honeybee (*Apis mellifera ligustica*) [44]. In these experiments 18 blue and 18 white feeders were used, randomly distributed within a 6×6 square array and the concentrations of the two feeder colors were systematically varied. There were 27 different concentration pairings (7 experiments×4 treatments minus 1 treatment from the first experiment) for which relative visitation rates to the higher concentration feeders for different sets of 3–4 bees over 40 visits per bee per treatment were measured. The mean sucrose concentrations in the seven experiments were from 0.25 to 1.75 M (8.3 to 49% w/w), with differences between the two feeder colors of either 0 M (0% w/w), 0.2 M (6.7% w/w), 0.4 M (13.0% w/w), or 0.6 M (19.1% w/w). Discrimination performance data were extracted from Figures III though IX, Chapter 4 in [44].

## Results

**Figure 2:**
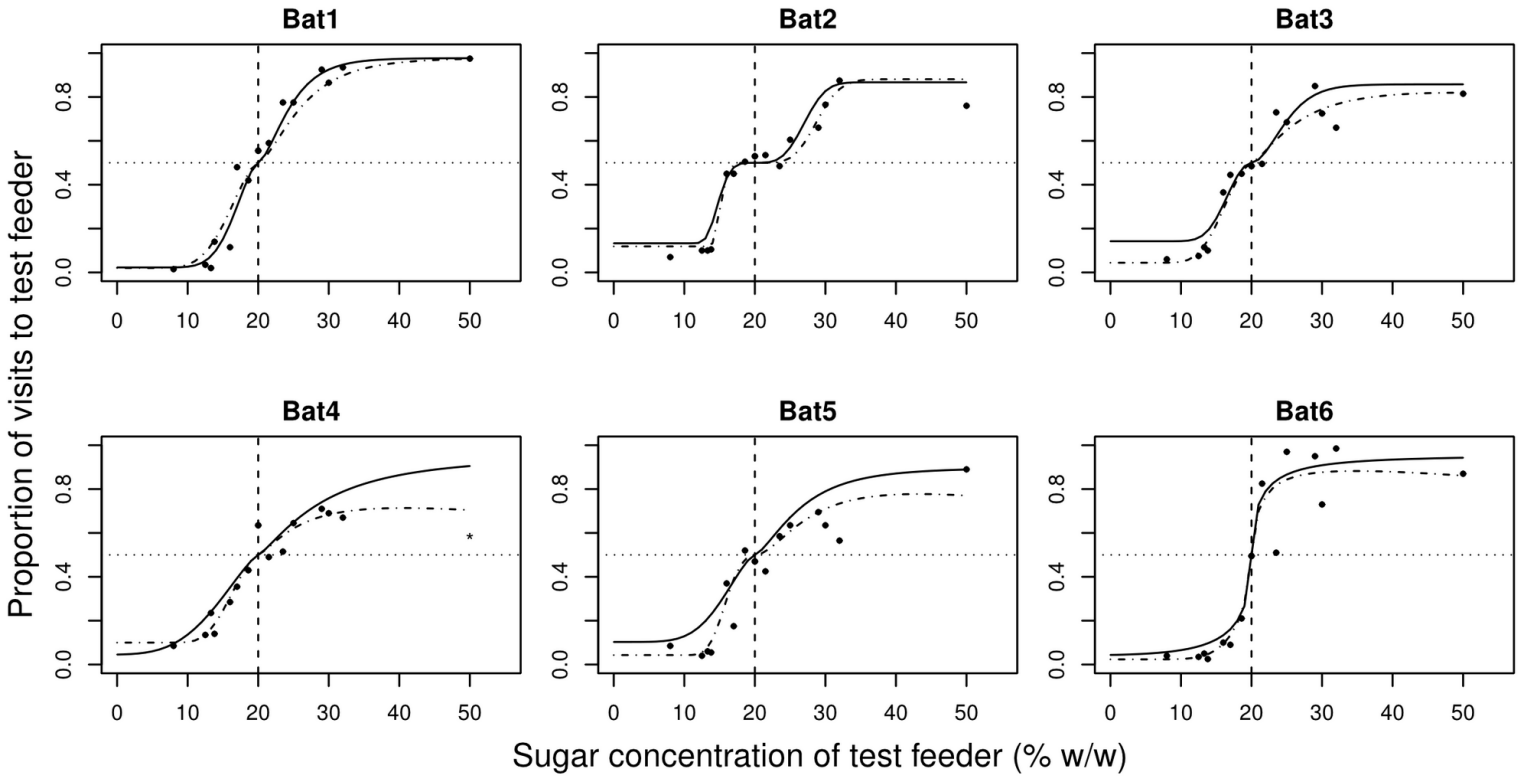
Discrimination performance at different concentrations of the test feeder for the six experimental subjects. The sugar concentrations of the test options are given on the abscissa. Black circles represent the proportion of visits to the test feeder averaged over the two presentations of the same pairs of concentrations (Table 1). The vertical dashed line indicates the standard option (20% w/w). The horizontal dotted line indicates the chance level at 0.5. Continuous lines give the non-linear fit based on Weber’s law model (Equation 5 with β = 1). Dash-dotted lines give the non-linear fit based on the near-miss to Weber’s law model (Equation 5 with β as a free parameter). The data point for Bat 4 at 50% sugar concentration (star) was excluded as an outlier.

**Figure 3:**
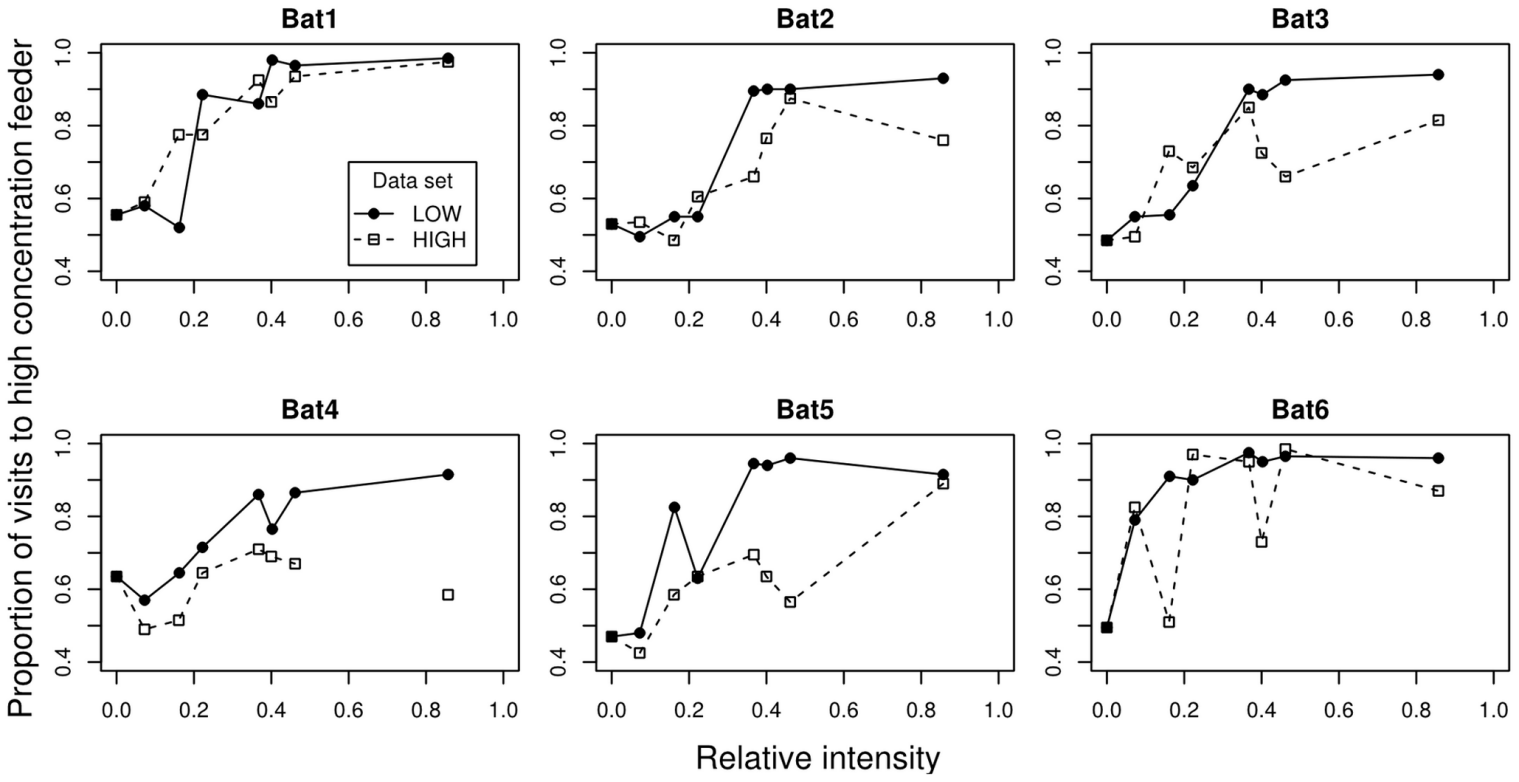
Discrimination performance as a function of relative intensity for the six experimental subjects. The same data as in Figure 2 are plotted, but with relative intensity on the abscissa (see Methods and Table 1). Black circles represent the proportion of visits to the standard feeder (with concentration of 20% w/w) in choices when the test feeder had a lower concentration than the standard (LOW data set). White squares represent the proportion of visits to the test feeder in choices when its concentration was higher than that of the standard (HIGH data set).

The discrimination performance of the bats varied with the magnitude of the test option. Bats either preferred the higher nectar concentration or showed no preference between the referent and standard feeders (Figure 2). Contrary to the prediction based on Weber’s law, expressing the differences between nectar concentrations in terms of relative intensity did not result in the same discrimination performances for the LOW and HIGH data sets in all animals (Figure 3). In the HIGH data set Bat 4 reached a maximum discrimination performance of 0.71 and at the highest intensity (*i* = 0.86) its discrimination performance actually dropped to 0.59. As these values resulted in high uncertainty of the psychometric function parameters, we excluded as an outlier the point at *i* = 0.86 from further analysis.

**Figure 4:**
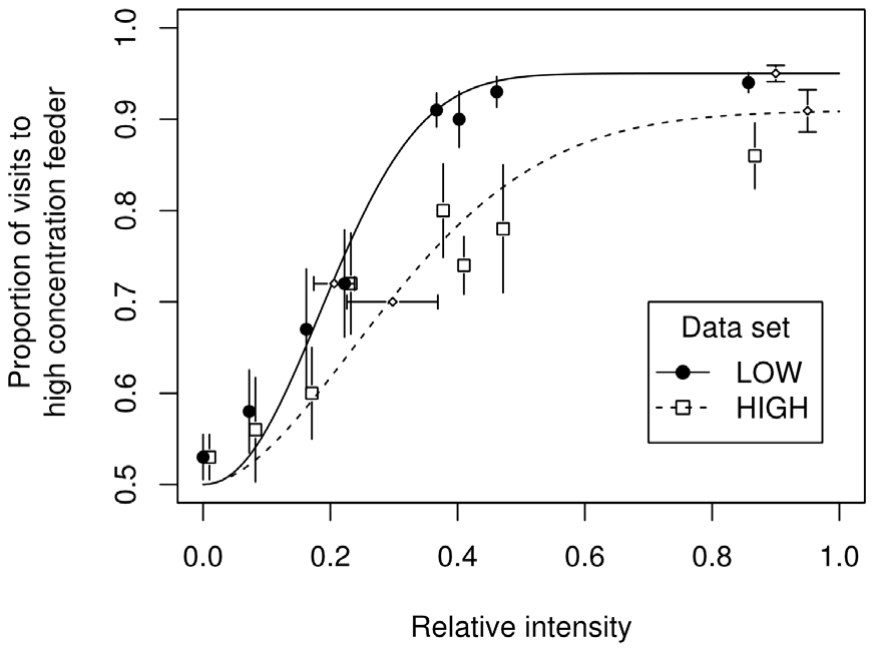
Psychometric curves for the LOW and HIGH data sets. The abscissa gives the relative intensity. Black circles represent the average proportion of visits to the standard feeder (with concentration of 20% w/w) over the six experimental animals in choices when the test feeder had a lower concentration than the standard (LOW data set). White squares represent the average proportion of visits to the test feeder over the six experimental animals in choices when the concentration of the test feeder was higher than that of the standard (HIGH data set). In order to prevent overlap in the graph, white squares are plotted with a horizontal jitter of 0.01 to the right. Vertical bars represent standard errors. The continuous curve represents the psychometric function with parameters (lapse rate, threshold and slope) averaged over the psychometric function parameters of the six experimental animals individually estimated from the LOW data set. The dashed curve represents the average psychometric curve obtained from the HIGH data set using the same procedure. The mean values of the threshold and upper asymptote for each curve are represented by white diamonds. Whiskers represent the standard errors.

The average psychometric functions of the experimental subjects were significantly different for the LOW and HIGH data sets (Figure 4). The slope of the psychometric function from the HIGH data set (mean ± SE = 2.10±0.39) was significantly shallower than the slope obtained from the LOW data set (3.65±0.41, Paired *t*(5) = 4.47, *p* = 0.007). The lapse rate from the HIGH data set (0.18±0.05) was higher than the lapse rate from the LOW data set (0.10±0.02), but this difference did not reach significance (Paired *t*(5) = -2.38, *p* = 0.06). The thresholds of the two psychometric functions were 0.30±0.07 and 0.21±0.03 for the HIGH and LOW data sets, respectively, and did not differ significantly (Paired *t*(5) = -1.42, *p* = 0.21). (With the outlier from the data set of Bat 4 included, the mean ± SE of the slope, lapse rate, and threshold of the HIGH data set were 1.93±0.49, 0.19±0.05, and 0.40±0.15, respectively. The resulting paired t test values for the comparison of the LOW and HIGH data sets were *t*(5) = 5.71, *p* = 0.002 for the slope, *t*(5) = -2.67, *p* = 0.04 for the lapse rate, and *t*(5) = -1.38, *p* = 0.23 for the threshold.).

For two of the six bats (Bats 1 and 6; Figure 2; Table 2) Weber’s law and the near-miss to Weber’s law resulted in equally good fits. For the remaining four bats the near-miss to Weber’s law was a significantly better model (Bats 2–5; Figure 2; Table 2). The estimated average value (± SE) for the exponent in the near-miss to Weber’s law was 2.44±0.37 and was larger than one in all six bats.

**Table 2:** Model comparison between near-miss to Weber’s law and Weber’s law.

	Near-miss to Weber’s law		Weber’s law		Model comparison		
	*β* a	AIC	*β* a	AIC	ΔAIC	F	*p*
Bat 1	−0.30 | 1.11 | 2.52	−32.59	1.00	−34.56	−1.97	0.022	0.885
Bat 2	0.54 | 1.81 | 3.57	−43.34	1.00	−35.71	7.63	9.901	0.008
Bat 3	1.53 | 2.37 | 3.22	−33.70	1.00	−28.86	4.83	6.393	0.026
Bat 4^b^	1.15 | 2.94 | 4.73	−31.47	1.00	−25.54	5.93	7.659	0.018
Bat 5	1.71| 2.72 | 3.72	−23.32	1.00	−14.30	9.02	11.893	0.005
Bat 6	0.14 | 3.70 | 7.25	−18.33	1.00	−18.16	0.17	1.747	0.211

In both models Equation 5 was fitted against observed individual discrimination performances. Lower Akaike Information Criterion (AIC) scores indicate a better fit of a model to the data, after penalizing for the number of estimated parameters. AIC scores can only be compared within rows but not between rows. ΔAIC gives the difference between the AIC scores for the model based on Weber’s law and the model based on the near-miss to Weber’s law. *F* and *p* values are based on one-way ANOVAs with 1 df.

a The exponent *β* was fixed with value one in the Weber’s law model and was estimated in the near-miss to Weber’s law model. Values in the middle are average estimates and the values to the left and right are the 95% confidence interval limits.

b One outlier was removed from the HIGH data set of Bat 4.

### Review of Sugar Discrimination in Different Nectar-feeding Animals

**Figure 5:**
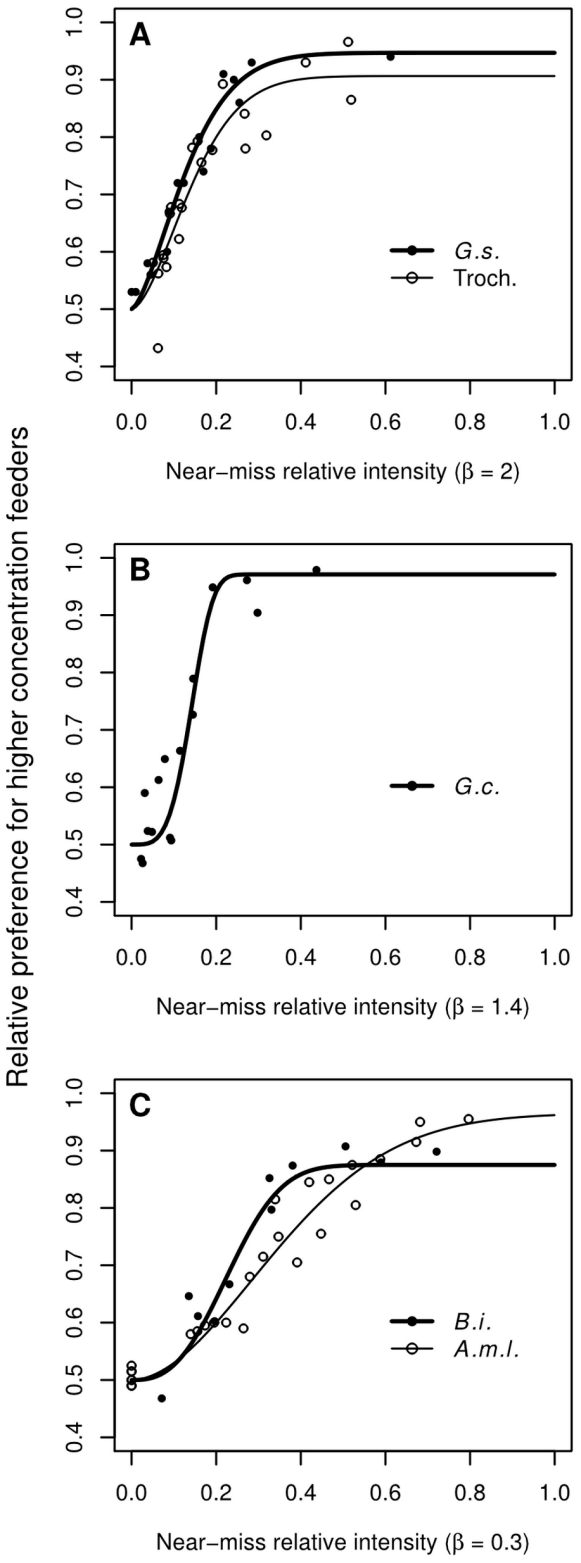
Psychometric curves based on near-miss relative intensities for different nectarivorous species. The abscissa gives the near-miss relative intensities as defined in equation (4) and scaled with different scaling factors. Symbols give average discrimination performances measured in different experiments with different species. Lines give the fitted psychometric functions. (**A**) Psychometric functions for hummingbirds (thin line, empty circles) and *G. soricina* (thick line, black circles). The exponent *β* in equation (4) was fixed at 2 and the scaling factor was 10. *G.s. – Glossophaga soricina*, this study; Troch. – different hummingbird (Trochilidae) species, [43]. (**B**) Psychometric function for *G. commissarisi* (thick line, black circles). The exponent *β* was fixed at 1.4 and the scaling factor was 1. *G.c. – Glossophaga commissarisi*, [23]; (**C**) Psychometric functions for bumblebees (thick line, black circles) and honeybees (thin line, empty circles). The exponent *β* was fixed at 0.3 and the scaling factor was 0.1. *B.i. – Bombus impatiens*, [24]; *A.m.l. – Apis mellifera ligustica*, [44].

In all of the analyzed data sets the estimates for the exponent *β* from Equation 5 statistically differed from one (Figure 5; Table 3). In the vertebrate group of nectar-feeding animals *β* was estimated at 2.39 in *G. soricina*, 1.43 in *G. commissarisi*, and 2.09 in hummingbirds (Table 3). The 95% confidence intervals for these estimates spanned 2.0 in *G. soricina* and in hummingbirds, but neither of these intervals overlapped with the confidence interval estimated in *G. commissarisi* (Table 3). Thus, for psychometric analyses, *β* was set at 1.4 in *G. commissarisi* and at 2.0 in *G. soricina* and in hummingbirds. In the bees the estimate for *β* was 0.29 in *A. mellifera ligustica* and -0.04 in *B. impatiens* (Figure 5; Table 3). The 95% confidence interval for *β* in *A. mellifera ligustica* did not span zero, but that of *B. impatiens* did. However, since both confidence intervals overlapped and spanned 0.3, in further psychometric analyses we set *β* at 0.3 in *A. mellifera ligustica* and in *B. impatiens*.

**Table 3:** Model comparison between near-miss to Weber’s law and Weber’s law in different nectar-feeding animals.

Species	Near-miss to Weber’s law		Model comparison with Weber’s law		
	*β* a	AIC	ΔAIC	F	*p*
*G. soricina*	1.81 | 2.39 | 2.99	−153.3	−14.6	17.3	***
*G. commissarisi*	1.29 | 1.43 | 1.58	−385.2	−28.0	31.2	***
Trochilidae	1.59 | 2.09 | 2.59	−58.2	−23.2	38.5	***
*B. impatiens*	−0.51 | −0.04 | 0.55	−66.6	−8.0	10.3	0.004
*A. mellifera ligustica*	0.15 | 0.29 | 0.43	−96.2	−31.3	55.8	***

In both models Equation 5 was fitted against observed discrimination performances. Lower Akaike Information Criterion (AIC) scores indicate a better fit of a model to the data, after penalizing for the number of estimated parameters. AIC scores cannot be compared between rows. ΔAIC gives the difference between the AIC scores for the near-miss to Weber’s law model and Weber’s law model. *F* and *p* values are based on one-way ANOVAs with 1 df.

Sources: *Glossophaga soricina*, this study; *Glossophaga commissarisi*, [23]; different Trochilidae species, [43]; *Bombus impatiens*, [24]; *Apis mellifera ligustica*, [44].

*** *p*<0.001.

a The exponent *β* was estimated in the near-miss to Weber’s law model and fixed at one in the Weber’s law model. Values in the middle are average estimates and the values to the left and right are the 95% confidence interval limits.

The estimates for the threshold *m*, slope *s*, and lapse rate *π_l_* for the psychometric functions of the different groups of animals were as follows: *G. soricina: m* = 0.12, *s* = 4.4, *π_l_* = 0.11; *G. commissarisi: m* = 0.14, *s* = 9.7, *π_l_* = 0.06; Trochilidae: *m* = 0.14, *s* = 4.3, *π_l_* = 0.19; *B. impatiens: m* = 0.23, *s* = 4.1, *π_l_* = 0.25; *A. mellifera ligustica: m* = 0.35, *s* = 1.9, *π_l_* = 0.07. The values for the lapse rates were in the range of 0.06–0.25 and were, as expected, somewhat higher than the typical lapse rates in human studies (0.0–0.10; [15]). The psychometric functions are shown in Figure 5.

## Discussion

The results from this study (Figures 2–4; Table 2) as well as the reanalysis of previously published data for different nectar-feeding animals (Figure 5; Table 3) all support the near-miss to Weber’s law as a better predictor of discrimination performance than Weber’s law. This means that when the options within two sets of alternatives differ by the same Weber fraction the probabilities of choice for each of the two options within one set of alternatives still changes as overall intensity increases. It is important to note exactly in which direction Weber’s law fails to predict discrimination performance in the different animal groups. As explained in the introduction, the near-miss to Weber’s law is a quantitative refinement of Weber’s law introduced as an attempt to correct for the overestimation of the magnitude effect when applying Weber’s law to data from acoustical discrimination tasks [9]–[12], [18]. In our review of sugar concentration discrimination in bees the magnitude effect was also found to be weaker than predicted by Weber’s law, since the exponent *β* was significantly lower than one (Table 3). However, the estimate for *β* was significantly higher than one in all vertebrates (Table 3). Thus, the magnitude effect in the vertebrates was actually stronger than predicted by Weber’s law.

The estimate for the *β* exponent for bumblebees was zero (Table 3), suggesting the absence of the magnitude effect. However, the 95% confidence interval was quite broad and included the value estimated for honeybees (0.3, Table 3). Furthermore, the sugar concentrations tested with bumblebees were only in the range of 15–50% w/w and were probably too high to allow the detection of the magnitude effect. In the full honeybee data set the range of sugar concentrations was broader (1.7–55.5% w/w). When the sessions with concentrations below 10% w/w were removed from the honeybee data set, the estimate for *β* was also reduced to zero (not shown). We tentatively conclude from this analysis that the magnitude effect in bees is small and only detectable when a broader range of sugar concentrations is tested, including values lower than 10%.

As mentioned in the introduction, the psychometric function in animal studies estimates discrimination performance rather than the capacity for perceptual discrimination. Animals might perceive differences between the available options but distribute their visits more evenly between the alternatives, regardless of expected value. If we challenge the assumption that the probability to lapse (i.e. make a visit at random) is constant and independent from the presented stimuli, an alternative explanation for differences in performance and the observed magnitude effect could be the trade-off between exploitation and exploration. In other words, animals might achieve perfect perceptual discrimination (for options that are sufficiently different) but lapse more often when the costs of information-gathering are low, i.e., when food resources are rich or when animals are at high energetic states. In *Drosophila* for example, appetitive memory performance has been demonstrated to decrease with satiety [45]. However, if a richer environment promoted lapsing, then the *G. commissarisi* bats from the field study should have lapsed more often in the 5% vs. 20% and in the 15% vs. 30% conditions when average total sugar reward was higher than in the 5% vs. 10% condition (assuming equal perceptual discriminability under all conditions). The observed lapse rates showed the opposite pattern and were the highest in the poorest condition (0.04 in the 5% vs. 20% condition, 0.10 in the 15% vs. 30% condition, and 0.20 in the 5% vs. 10% condition [23]). The variable lapse rate hypothesis cannot be discarded based on this counter-argument, but we consider it a less likely explanation for the observed patterns of discrimination performance.

In the remainder of this section we will discuss the differences in discrimination performance between the different groups of nectar-feeding animals and relate these differences to the nectar traits of plants pollinated primarily by vertebrate or by bee pollinators. Typical bat-pollinated and hummingbird-pollinated plants have dilute nectars with sugar concentrations of 13–18% w/w [46], [47] and 23% w/w [46], respectively. Typical bee-pollinated plants on the other hand tend to have nectars with higher sugar concentrations [46]. On the evolutionary timescale, bees and bee-pollinated plants predate vertebrate pollinators [48] and transitions from insect pollination to vertebrate pollination are more common than vice versa [49], [50]. Based on these observations, it appears that transitions from bee to vertebrate pollination are associated with a decrease in nectar sugar concentration. A reasonable expectation is therefore that discrimination performance for sugar concentration may be different in bees and in vertebrates, with bees possibly being better at discriminating between higher concentrations. In general, better discrimination performance can be indicated by a lower lapse rate, lower threshold, and steeper slope. Next, we consider each of these three psychometric function parameters in turn.

**Figure 6:**
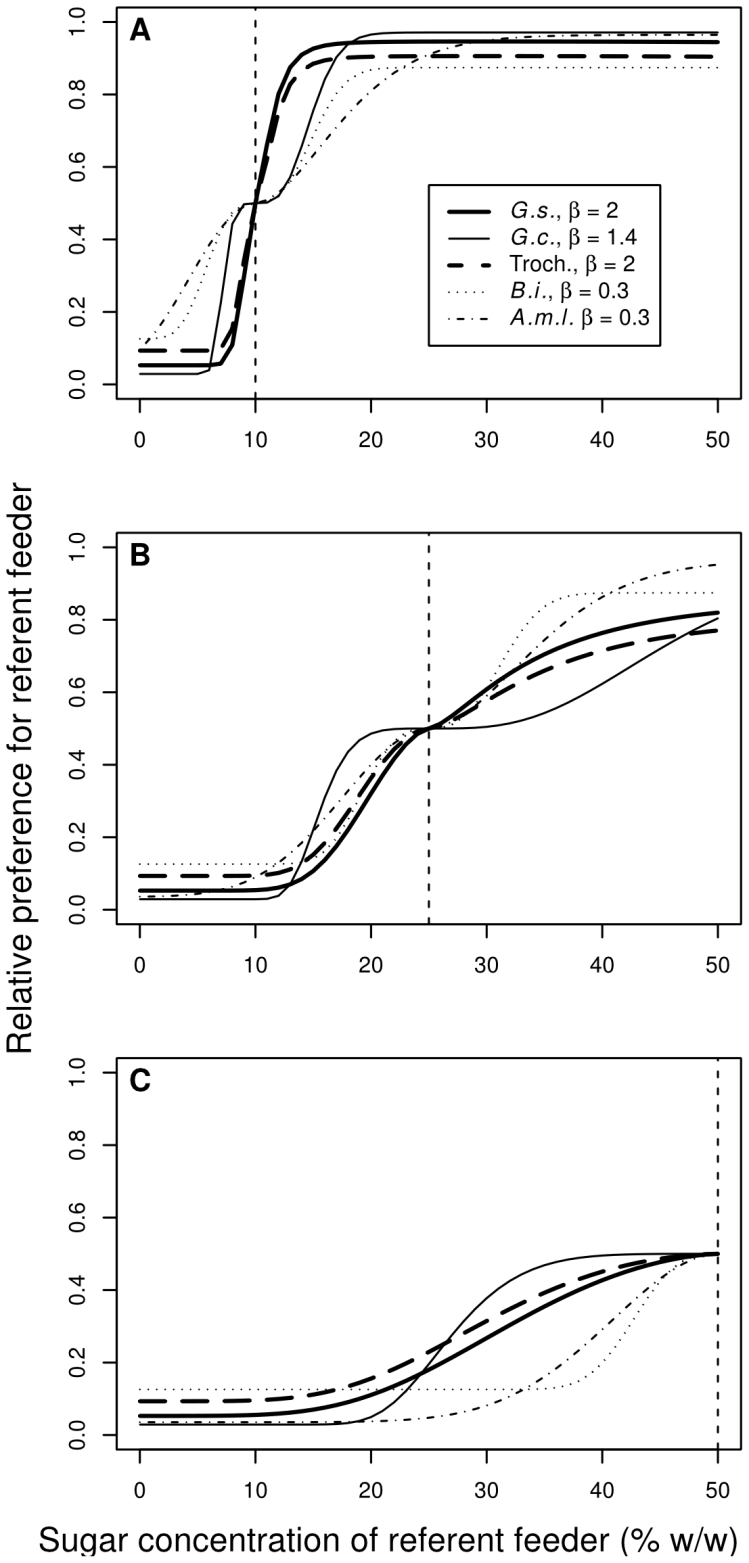
Predicted relative preference in choices between a standard option and alternatives with different sugar solutions. The vertical dashed line passes through the sugar concentration of the standard option in each panel. Perfect discrimination performance would look like a step function at zero before and at one after the sugar concentration of the standard. The closer a curve lies to the vertical dashed line, the better the predicted discrimination performance. (**A**) Standard option with 10% w/w concentration. (**B**) Standard option with 25% w/w concentration. (**C**) Standard option with 50% w/w concentration. In each panel different lines (see legend in A) give the predicted preferences for the alternative option of different nectar-feeding species: *G.s. – Glossophaga soricina*, this study; *G.c. – Glossophaga commissarisi*, [23]; Troch. – different hummingbird (Trochilidae) species, [43]; *B.i. – Bombus impatiens*, [24]; *A.m.l. – Apis mellifera ligustica*, [44]. The values of the exponent *β* are given in the legend. Note that in A, in the concentration range 0–20% the insects have a higher probability of choosing the energetically less profitable option (i.e. choosing 5% instead of 10% or choosing 10% instead of 15%) than the vertebrates. However, in C the insects are much better at avoiding the options with less than 50% sugar.

The similar lapse rates in the different groups of animals suggest similar general motivational and explorative tendencies. As the lapse rates are fairly low, the psychometric functions in all animals are likely to give good approximations of the actual capacity for perceptual discrimination. The somewhat higher lapse rate in *B. impatiens* (*π_l_* = 0.25) was probably overestimated because of the lack of sessions with very low concentrations. The threshold and slope can only be directly compared in groups with the same *β* estimate. As detailed in the introduction, *β* is the parameter that determines how strong the magnitude effect is with respect to the distance effect. Such comparison was possible between *G. soricina* and hummingbirds with *β* = 2 and between honeybees and bumblebees with *β = *0.3 (assuming that the true value of *β* is similar in honeybees and bumblebees and that it was better estimated in *A. mellifera ligustica*). *G. soricina* had a psychometric function with a lower threshold and a steeper slope than the hummingbirds (Figure 5A). This difference is consistent with bats often visiting flowers with even more dilute nectars than hummingbirds. However, the discrimination performance of hummingbirds might have been underestimated because performance was scored as food intake rather than as asymptotic visitation rates. It has been demonstrated that the inclusion of the learning phase can shift the psychometric curve to the right and flatten it [17]. Furthermore, the psychometric functions for *G. soricina* and for the hummingbirds were more similar to each other than either of them was to the function fitted for *G. commissarisi* (Figures 5A and 5B; Figure 6). The poorer discrimination performance of *G. commissarisi* could be due to the higher difficulty of the task, in which 24 feeders rather than two were available. In the bee group the psychometric function of the bumblebees had a lower threshold and a steeper slope than that of the honeybees (Figure 5C). Again, the poorer performance of honeybees might be explained by the inclusion of the learning phase in the measure for discrimination performance. Nonetheless, the two functions were fairly similar (Figure 5C).

Using Equation 5 with the appropriate fitted parameters for each group it is possible to extrapolate discrimination performances of different nectar-feeding animals in 2AFC tasks for a given standard option. This allows us to compare discrimination performances for groups with different *β* parameters (Figure 6). Bats and hummingbirds are predicted to outperform bees when the standard option is at 10% w/w and the referent option is either more dilute or more concentrated than the standard (Figure 6A). In contrast, when the standard option is at 50% w/w and the referent option is lower than 50% w/w, bees are expected to outperform vertebrate nectar-feeding animals (Figure 6C). The situation is intermediate with a standard at 25% w/w concentration; all animals are expected to be about equally good at discriminating referent options with concentrations lower than 25% w/w, but bees are expected to outperform bats and hummingbirds if the concentration of the referent option is higher than 25% w/w (Figure 6B).

Thus, if Equation 5 accurately predicts discrimination performances, then bees do not simply outperform vertebrates. Instead the relative discrimination performance of different species is context-dependent: with high standards bees outperform vertebrates and with lower standards, vertebrates outperform bees. The mathematical explanation for this unexpected prediction lies in the strength of the magnitude effect (the value of *β*). Since the magnitude effect is much stronger in vertebrates, their initially better discrimination performance deteriorates faster with the increase in mean concentration, falling below the discrimination performance of bees. In summary, pollinators examined in this study are most sensitive to differences in sugar concentration in the typical ranges of the flowers they naturally pollinate.

The independently obtained estimates for the strength of the magnitude effect were similar within and different between groups of animal pollinators, suggesting that the small magnitude effect in bees and large magnitude effect in vertebrates may be the result from phylogenetic or morphological constraints. We expect the beta estimates for yet untested insect and vertebrate pollinators to align to the pattern suggested by our analysis. On the other hand, we expect that as nectarivores become more specialized, there is directional selection pressure for the threshold of their psychometric function for sugar discrimination to become even smaller and for the slope to become steeper. These hypotheses can be tested by subjecting more taxa of nectar-feeding animals to phylogenetic analyses of psychometric function parameters [51].

In conclusion, psychometric analyses such as the one presented here can be a useful tool for revealing expected differences in discrimination performance between different nectar-feeding animals. However, the predictive power of the near-miss to Weber’s law models needs to be verified empirically.

## Supporting Information

Table S1 **Pre-test conditions for the first group of subjects.**
(DOC)
