# Supplementary material for: Weber’s Law, the Magnitude Effect and Discrimination of Sugar Concentrations in Nectar-Feeding Animals
Source: PLoS One. 2013 Sep 10;8(9):e74144. doi: 10.1371/journal.pone.0074144 (PMC3769339; doi:10.1371/journal.pone.0074144)
Supplement: Table S1 — Pre-test conditions for the first group of subjects. (DOC) [file pone.0074144.s001.doc]

**Table S1. Sequence of experimental conditions in the first six nights of the first group of subjects (*N*** = 3 bats)

| Sequence^a^ | Test concentration^b^ | Relative intensity^c^ | Discrimination performance ± s.e.^d^ |
| --- | --- | --- | --- |
| 1 | 20 | 0.00 | 0.55 ± 0.07 |
| 2 | 16 | 0.22 | 0.61 ± 0.10 |
| 3 | 50 | 0.86 | 0.70 ± 0.11 |

These conditions immediately preceded the conditions given for Group 1 in Table 1. On nights 1-4 and on night 6, the same procedure was used as in the main experiment, except that after each visit during the choice phase, the two feeders were closed and remained inaccessible for 30 seconds. This step in the procedure lengthened the duration of the choice phase unnecessarily and was dropped in further experiments.

^a^ Each condition in the sequence was tested twice on two consecutive nights, with the position of the test and standard feeder exchanged.

^b^ Sugar solution concentrations are given in % weight/weight. The concentration of the standard was always 20% w/w.

^c^ Relative intensity is calculated as the absolute difference between the test and standard concentrations divided by the average of the concentrations.

^d^ Discrimination performance was averaged over the two presentations of the same condition for each of the three bats, then the grand mean for the respective condition was calculated. The standard error was calculated over the mean values of all bats.
